# Chronic Stressors and Adolescents’ Externalizing Problems: Genetic Moderation by Dopamine Receptor D4. The TRAILS Study

**DOI:** 10.1007/s10802-017-0279-4

**Published:** 2017-03-30

**Authors:** Anna Roos E. Zandstra, Johan Ormel, Pieter J. Hoekstra, Catharina A. Hartman

**Affiliations:** Department of Psychiatry, University of Groningen, University Medical Center Groningen, P.O. Box 30.001, 9700 RB Groningen, The Netherlands

**Keywords:** Chronic stressors, Psychosocial adversity, Sensitivity to the environment, Dopamine D4 receptor 7-repeat allele (*DRD4*–7R), Externalizing problems, Adolescence

## Abstract

The existing literature does not provide consistent evidence that carriers of the Dopamine D4 Receptor 7-repeat allele are more sensitive to adverse environmental influences, resulting in enhanced externalizing problems, compared to noncarriers. One explanation is that the adverse influences examined in prior studies were not severe, chronic, or distressing enough to reveal individual differences in sensitivity reflected by *DRD4*–7R. This study examined whether the 7-repeat allele moderated the association between chronic stressors capturing multiple stressful aspects of individuals’ lives and externalizing problems in adolescence. We expected that chronic stressor levels would be associated with externalizing levels only in 7-repeat carriers. Using Linear Mixed Models, we analyzed data from 1621 Dutch adolescents (52.2% boys), obtained in three measurement waves (mean age approximately 11, 13.5, and 16 years) from the TRacking Adolescents’ Individual Lives Survey (TRAILS) population-based birth cohort and the parallel clinic-referred cohort. Across informants, we found that higher levels of chronic stressors were related to higher externalizing levels in 7-repeat carriers but not in noncarriers, as hypothesized. Although previous studies on the 7-repeat allele as a moderator of environmental influences on adolescents’ externalizing problems have not convincingly demonstrated individual differences in sensitivity to adverse environmental influences, our findings suggest that adolescent carriers of the Dopamine D4 Receptor 7-repeat allele are more sensitive to chronic, multi-context stressors than noncarriers.

**Electronic supplementary material:**

The online version of this article (doi:10.1007/s10802-017-0279-4) contains supplementary material, which is available to authorized users.

Exposure to psychosocial stressors increases adolescents’ risk of psychopathology (for an overview, see Grant et al. 2004), including rule-breaking and aggressive (externalizing) behavior as seen in oppositional defiant disorder (ODD) and conduct disorder (CD). However, individual differences in outcome are large (Jenkins 2008; Rutter 2005), suggesting that some individuals are more sensitive to their environment than others.

A polymorphism in the third exon of the Dopamine D4 Receptor (*DRD4*) gene encodes for a variable number of tandem repeats, ranging from 2 to 11 (Bakermans-Kranenburg and Van IJzendoorn 2011; Dmitrieva et al. 2011; Ptacek et al. 2011). The 7-repeat (7R) variant results in lower affinity for dopamine (Ptacek et al. 2011), one of the brain’s chemical messengers that is of interest in relation to externalizing problems, through its assumed role in reward mechanisms, motivation, and approach behavior (Dmitrieva et al. 2011). The global frequency of the 7R allele is about 20%, with considerable variation across populations (Chang et al. 1996).

*DRD4*–7R has been extensively examined as a moderator of the association between environmental influences and externalizing problems, based on the notion that the 7R allele may reflect sensitivity to the environment, for better and for worse. These studies included influential, relatively proximal environmental factors (and not, for example, exposure to media or video game violence, see Ferguson 2015; Savage and Yancey 2008). According to this Differential Susceptibility model (e.g., Ellis et al. 2011), sensitive individuals are likely to be positively affected by beneficial environmental influences (e.g., peer acceptance) and negatively by adverse influences (e.g., psychosocial stressors), whereas less sensitive individuals are less affected by both.

Most empirical support for this model comes from studies on laboratory-observed parenting factors in relation to externalizing problems in toddlers and preschoolers. Specifically, 10 month-old 7R carriers exposed to low vs. high laboratory-observed maternal sensitivity showed high vs. low externalizing levels, respectively, approximately 2.5 years later; whereas noncarriers appeared unaffected by maternal sensitivity (Bakermans-Kranenburg and Van IJzendoorn 2006). In addition, an intervention aimed at reducing toddlers’ externalizing problems by promoting maternal positive discipline proved to be more effective in 7R carriers than in noncarriers at follow-up (mean age 27, 39, and 52 months at pretest, posttest, and follow-up, respectively, Bakermans-Kranenburg et al. 2008). However, one study showed that in European-Americans, the influence of warm-responsive and negative-intrusive parenting at 6 and 12 months on externalizing levels at 18, 24, and 30 months did not significantly differ between 7R carriers and noncarriers (Propper et al. 2007). Another study showed that maternal sensitivity at 14 months, but not at 36 or 48 months, interacted with *DRD4*–7R in predicting later externalizing problems (Windhorst et al. 2015). Specifically, higher maternal sensitivity at 14 months predicted lower externalizing levels at 18 months (as well as at 60 months, but only via indirect paths across time) in 7R carriers, but did not affect noncarriers. At 36 months, however, 7R carriers showed a similar response, but noncarriers showed the opposite (i.e., higher sensitivity predicted higher externalizing levels), rather than no response. One study in middle childhood showed higher sensitivity for better and for worse in 7R carriers compared to noncarriers, to verbal but not physical peer victimization and with respect to self-reported but not parent-reported externalizing problems (DiLalla et al. 2015). Thus, these prior findings do not consistently support the Differential Susceptibility model.

In samples with a broad age range that included adolescence findings showed no evidence that *DRD4*–7R moderated the influence of maternal expressed emotion (i.e., warmth, criticism) on conduct problems (mean age 11 years, range 5–17 years, Sonuga-Barke et al. 2009) or on prosocial and antisocial behavior (mean age 17 years, range 7–28 years, Richards et al. 2015). In studies that focused on externalizing problems in (pre)adolescents (between 11 and 20 years old), the notion that *DRD4*–7R may reflect sensitivity to the environment, for better and for worse, has received moderate but inconsistent support. One study showed higher sensitivity, for better and for worse, in 7R carriers, compared to noncarriers; to laboratory-observed early maternal stimulation and responsiveness, but not to parent-reported early family adversity, with respect to adolescents’ symptoms of CD/ODD (combined parent-report and self-report) and psychopathy (parent-report, Nikitopoulos et al. 2014). Another study showed relatively high sensitivity, for better and for worse, in 7R carriers to a broad range of intervention-targeted parenting behaviors (reported by parents), with respect to self-reported substance use, but not to parent-reported delinquency (Beach et al. 2010). In addition, recent findings showed higher sensitivity for better and for worse in 7R carriers compared to noncarriers, to positive and negative social preference with respect to teacher-reported conduct problems (Buil et al. 2015). In contrast, no moderating effect of *DRD4* genotype was found on the association between peer influence (self-reported peer rejection and acceptance) and several measures of externalizing problems (parent-report and self-report, Janssens et al. 2015).

Prior studies from our research group TRAILS (TRacking Adolescents’ Individual Lives Survey) have, likewise, produced inconsistent results. One of these (Nederhof et al. 2012a) showed that the 7R allele moderated the association between parental separation and self-reported externalizing problems, although this effect pertained only to boys, not to girls, and only to the absence of parental separation, not to its presence. That is, externalizing levels of 7R–carrying boys compared to noncarriers were relatively low if their families were intact but did not differ if their parents had separated, suggesting sensitivity for better but not for worse. Other studies from our research group showed no evidence that 7R carriers are relatively sensitive to the influence of peers (teacher-reported peer victimization and self-reported social well-being) on self-reported delinquency (Kretschmer et al. 2013) or of parenting (rejection, overprotection, and emotional warmth as reported by pre-adolescents) on delinquency and aggression (combined parent-report and self-report, Marsman et al. 2013) or substance use (self-report, Creemers et al. 2011).

Taken together, there are clearly many inconsistencies in the literature as to whether *DRD4*–7R may reflect individual differences in sensitivity to environmental influences. The inconsistencies in prior findings in adolescence, which is also the focus of the present study, do not appear to be driven by an informant effect, nor by differences in operationalization of externalizing problems (e.g., substance use vs. delinquency vs. broader externalizing measures) or environmental influences (e.g., parent vs. peer influence; broad vs. narrow aspects of parenting). Rather, what seems to stand out in these prior findings is the lack of evidence that *DRD4*–7R reflects sensitivity to the detrimental effects of adverse environmental influences. Of the few findings that did support high sensitivity not only for better but also for worse in 7R carriers (cf. Beach et al. 2010; Buil et al. 2015; Nikitopoulos et al. 2014) most were based on the absence of positive (beneficial) environmental influences. For example, while high levels of maternal stimulation and responsivity in the study by Nikitopoulos et al. (2014) were considered to be beneficial, low levels reflect an absence of beneficial influence, rather than presence of adverse influence (e.g., the presence of maternal hostility). In contrast, of the prior findings in adolescence relating to actual adverse influence (i.e., early family adversity, perceived parental rejection or overprotection, parental divorce or separation, peer victimization, peer rejection, negative social preference) only one (Buil et al. 2015) suggested differences in externalizing levels between 7R carriers and noncarriers (Creemers et al. 2011; Janssens et al. 2015; Kretschmer et al. 2013; Marsman et al. 2013; Nikitopoulos et al. 2014). Thus, in adolescents, the Differential Susceptibility hypothesis, extending the Diathesis-Stress theory (Zuckerman 1999) that some individuals are more vulnerable to the detrimental effects of adverse influences, has not received much support from the data.

One explanation could be that the adverse environmental influences examined in prior studies were not severe, chronic, or distressing enough to reveal individual differences in sensitivity reflected by *DRD4*–7R. Adverse environmental influences may become more severe or distressing as they persist over time, taxing individuals’ physical and psychological coping resources. In addition, subtle individual differences in sensitivity may be missed when the adverse environmental influence is rather narrowly operationalized, capturing only one aspect of individuals’ lives. That is, the adverse environmental influences examined in prior studies generally reflected a specific aspect of a single environmental domain (e.g., either family or peer group) while beneficial influences from other domains, if present, will compensate for their impact. Individual differences in sensitivity may thus be easier to detect by assessing environmental influences that are chronic and reflect multiple adverse aspects across multiple environmental domains (e.g., family, peers, school, and neighborhood). We hypothesize that if *DRD4*–7R truly reflects individual differences in sensitivity to the environment, not only for better, as some prior findings have shown, but also for worse, this may become evident in the presence of chronic, multi-context stressors, which may exceed sensitive individuals’ ability to cope.

This study aimed to enhance our understanding of individual differences in adolescents’ externalizing problems following exposure to chronic stressors. To this end, we have examined whether *DRD4*–7R is a moderator of the association between chronic stressors, operationalized as number of long-term difficulties, and externalizing (CD and ODD) problems from preadolescence into adolescence. We expected that, only in 7R carriers, chronic stressor levels would be positively associated with externalizing levels, whereas in noncarriers, we expected no influence of chronic stressors on externalizing levels.

## Method

### Participants

We obtained the data used in this study from the first three measurement waves of TRAILS (mean ages about 11, 13.5, and 16 years). TRAILS aims to contribute to the understanding of the etiology of mental health problems by following 10–12 year-old Dutch children biennially into adulthood.

We pooled data from the TRAILS population-based birth cohort (*n* = 2230) and the parallel clinic-referred cohort (*n* = 543), to obtain a large sample with a wide range of problem severity and chronic stress. The sampling procedures, descriptive statistics, and response rates of both cohorts are well-documented (e.g., De Winter et al. 2005; Huisman et al. 2008; Ormel et al. 2012). In brief, TRAILS approached 135 primary schools in five municipalities in the Northern Netherlands to build the population cohort. Of these schools, 90.4% agreed to participate. TRAILS contacted eligible students and their parents (excluding individuals with mental retardation and individuals without a Dutch-speaking parent or parent surrogate), enrolling 76% (*n* = 2230; 49.2% boys; 86.5% Dutch ancestry; mean age 11.11; *SD* 0.56; range 10.01–12.58) of those contacted in the study. The three data waves we included in this study ran from March 2001 to July 2002 (T1), September 2003 to December 2004 (T2), and September 2005 to August 2007 (T3); with response rates consistently above 80%.

The smaller clinic-referred sample (*n* = 543) consists of pre-adolescents who had been referred to the Groningen University Child and Adolescent Psychiatric Outpatient Clinic at any point in their life (20.8% ≤5 years; 66.1% 6–9 years; 13.1% 10–12 years) for consultation or treatment. The first three data waves in the clinic-referred cohort ran two years behind those of the population cohort: From September 2004 to December 2005 (T1), September 2006 to November 2007 (T2), and September 2009 to February 2011 (T3). The measurement instruments and design for the clinic-referred cohort were the same as those of the population cohort. Of the 1264 eligible pre-adolescents, 543 (65.9% boys; 98.2% Dutch ancestry; mean age 11.11; *SD* 0.50; range 10.13–12.40) enrolled in the study and finished baseline measurements (T1). Of these 543 baseline participants, 85.1% (*n* = 462) participated in the second wave (T2). Of the T2 participants, 83.5% (*n* = 386) also participated in the third wave (T3). Another 30 T2 dropouts agreed to participate in the third wave, resulting in a total T3 response rate of 76.6% (*n* = 416) of the original sample. Selective attrition analyses have been described elsewhere (De Winter et al. 2005; Huisman et al. 2008; Nederhof et al. 2012b; Ormel et al. 2012). Importantly, baseline participants did not differ from non-participants with respect to externalizing problems.

Compared to the population cohort, the clinic-referred cohort had, on average, a higher socio-economic status, *t* (886.310) = 4.548, *p* < 0.001, consisted of more boys, *t* (859.668) = 7.274, *p <* 0.001, and less individuals of non-Dutch ancestry, *t* (2209.170) = 12.563, *p* < 0.001.

### Procedures

Every measurement wave, adolescents and their parents (typically the mother, >95%) filled out several questionnaires. Parents were assessed at home. Adolescents were assessed at school (population cohort) or at the Groningen University Child and Adolescent Outpatient Clinic (clinic-referred cohort), under the supervision of one or more well-trained assistants. For adolescents’ DNA analysis, blood or buccal cells were collected at T2 (clinic-referred cohort) or T3 (population cohort). Parents gave written informed consent prior to each assessment wave. Adolescents gave written informed assent at the second and third wave. TRAILS was approved by the National Dutch Medical Ethics Committee, in accordance with the ethical standards laid down in the 1964 Declaration of Helsinki.

### Measures

#### Externalizing Problems

TRAILS used the Achenbach System of Empirically Based Assessment (ASEBA) family of measures of mental health problems (Achenbach and Rescorla 2001; Verhulst and Van der Ende 2013) at each time point. The Child Behavior Checklist (CBCL) and the Youth Self-Report (YSR) contain 120 items assessing behavioral and emotional problems in children over the past 6 months. These items can be rated as 0 *(not true),* 1 (*somewhat or sometimes true),* or 2 (*very or often true)*. We used DSM-IV-oriented subscales to define externalizing problems as the sum of the average scale scores of oppositional defiant problems (*k* = 5; Cronbach’s α = 0.81 and α = 0.64 for parent-report and self-report, respectively) and conduct problems (*k* = 17, α = 0.82 for parent-report; *k* = 15, α = 0.75 for self-report). Then, sum scores were standardized.

Externalizing problems correlated significantly (*p <* 0.001) with internalizing problems, at both T2 (*r* = 0.52, parent-report, and *r* = 0.38, self-report) and T3 (*r* = 0.54, parent-report, and *r* = 0.32, self-report). Since internalizing problems have no theoretical and empirical relevance in relation to *DRD4*–7R (e.g., Bakermans-Kranenburg and Van IJzendoorn 2006; DiLalla et al. 2015), potential interaction effects of *DRD4*–7R and stressors in predicting externalizing problems may be weakened by the presence of co-occurring internalizing problems. Therefore, we focused on externalizing problems adjusted for co-occurring internalizing problems (EXTadj). To that end, we computed the summed weighted average of anxiety (*k* = 6; α = 0.73 and α = 0.61 for parent-report and self-report, respectively) and affective problems (*k* = 13; α = 0.72 and α = 0.71 for parent-report and self-report, respectively), after which we computed residual externalizing scores (*M* = 0; *SD* = 1). Although our main focus is on residual externalizing problems (EXTadj), results are also described for unadjusted externalizing problems (EXT), with and without correcting for internalizing problems as a covariate.

#### Chronic Stressors Preceding T2 and T3

We operationalized chronic stressor levels at T2 and T3 as the number of parent-reported long-term difficulties since the previous measurement. One of the parents, typically the mother, filled out a TRAILS questionnaire that listed long-term difficulties that were described in a broad way in an effort to capture multiple possible subtypes to which the adolescent might have been exposed since the previous interview (e.g., Oldehinkel et al. 2008; Zandstra et al. 2015). The stressors included: (1) chronic illnesses or physical handicaps of the child or (2) a family member; (3) high work pressure at school; (4) housing problems; (5) neighborhood problems, such as violence or discrimination; (6) financial problems; (7) lack of friends; (8) being bullied; (9) long-lasting conflicts with family members or (10) others; and (11) long-lasting conflicts between family members. On an open item, parents could also disclose additional long-term difficulties. We coded these additional problems either as a long-term difficulty or dismissed them according to well-defined rules—in particular whether the described situation is typically considered stressful and enduring. For example, we coded a turbulent home environment, such as moving frequently from house to house or parents having an on/off relationship, as long-term difficulties. Situations that we rejected as long-term difficulty included normative or non-enduring situations such as the transition to middle school, puberty, and quarrels with siblings. The number of reported difficulties ranged from 0 to 10. To reduce the influence of extreme and rare scores, we truncated 3 to 10 long-term difficulties as 3 or more, based on the frequency distribution (see Online Resource 1).

#### DRD4 Genotyping

DNA was extracted from blood samples or buccal swabs (Cytobrush®) using a manual salting out procedure (Miller et al. 1988). The 48 bp direct repeat polymorphism in exon 3 of *DRD4* was genotyped on the Illumina BeadStation 500 platform (Illumina Inc., San Diego, CA, USA), described in detail elsewhere (Nederhof et al. 2012a). The genotyping assay was carried out in a CCKL quality-certified laboratory and has been validated in earlier tests. Three percent blanks as well as duplicates between plates were processed as quality controls during genotyping. Determination of the length of the alleles was performed by direct analysis on an automated capillary sequencer (ABI3730, Applied Biosystems, Nieuwerkerk, The Netherlands) using standard conditions. We formed two groups according to the presence of at least one 7R allele (1 = 7R carrier; 0 = noncarrier, i.e., all others).

### Data Analysis

#### Data Preparation and Preliminary Analyses

For this study, our statistical analysis method required at least one value for each predictor on T1-T3 and at least T2 or T3 externalizing problems. Thus, we needed T2 and/or T3 parent-reported and/or self-reported externalizing problems, T2 and/or T3 chronic stressors, and *DRD4*. Participants not from Dutch ancestry were excluded, since genetic effects and gene-environment interaction effects are not necessarily generalizable across racial populations (Bakermans-Kranenburg and Van IJzendoorn 2011; also see Propper et al. 2007). Of each sibling pair, we excluded one participant at random. We performed independent samples *t-*tests to check whether included and excluded subjects differed with respect to our study variables.

#### Main Analyses

We computed correlation coefficients between the predictors and T2 and T3 externalizing problems. The possible presence of gene-environment correlations (i.e., *DRD4* genotype is associated with exposure to chronic stressors) may drive gene-environment interaction effects and therefore needs to be ruled out.

We used Linear Mixed Modeling (LMM) to investigate the effects of chronic stressors, *DRD4*–7R, and their hypothesized interaction in predicting subsequent EXTadj. LMM allows for missing data at different measurement waves, which is an important advantage for a longitudinal design (Kwok et al. 2008). Using PASW Statistics 18, we conducted LMM analyses (T2 and T3 in a single analysis), separately for parent-reported and self-reported EXTadj. We included the independent variables of age (time-variant), sex (0 = female; 1 = male), initial EXTadj at T1, chronic stressors (time-variant), and *DRD4*–7R, as well as an interaction between chronic stressors and *DRD4*–7R. All non-dichotomous variables were centered prior to analysis. For interpretation of interaction effects we plotted EXTadj levels based on the estimated regression coefficients, for different levels of each predictor. We used the Maximum Likelihood estimation procedure and considered a *p*-value < 0.05 to be statistically significant.

For post-hoc probing of statistically significant interaction effects, we computed simple slopes, which reflect the slopes of regression lines in a plot, and regions of significance, indicating the range of values of a predictor at which the interaction effect is statistically significant (Preacher et al. 2006). Regions of significance result from separate analyses that may produce values of a predictor that fall outside the true data range. To examine the potential influence of sex on significant interaction effects, we repeated our main analyses (in which we controlled only for a main effect of sex on EXTadj) by adding sex by *DRD4*–7R and sex by stressors interaction terms to the model, as has recently been recommended in the literature (Keller 2014). If findings showed significant sex by predictor interaction effects, we tested for an additional three-way interaction effect of chronic stressors, *DRD4*–7R, and sex, in predicting EXTadj. To check the influence of co-occurring internalizing problems, we repeated the analysis replacing the outcome variable EXTadj with EXT; that is, externalizing problems unadjusted for co-occurring internalizing problems. Additionally, we added internalizing problems as a covariate to the model, to rule out the possibility that our main findings are driven by the use of residual externalizing problems.

## Results

### Results of Preliminary Analyses

Three hundred and nine participants had missing data for both measurements of chronic stressors, and 137 for parent-reported as well as self-reported externalizing problems. Of the 1861 participants with available *DRD4* data, those not from Dutch ancestry (*n* = 166) were excluded. Of the sibling pairs in the remaining groups, one of each was excluded (*n* = 22). Altogether, we excluded a total of 1152 participants (*n* = 1005 population cohort; *n* = 147 clinic-referred cohort) from this study, resulting in a final sample of 1621 subjects (*n* = 1225 population cohort; *n* = 396 clinic-referred cohort).

We compared the final study sample (mean age 11.09; *SD* 0.55; range 10.01–12.58; 52.2% boys; 75.6% population cohort) with those who were not included. We found that participants were somewhat younger, *t* (2769) = −2.309, *p* = 0.021, and had higher T2 chronic stressor levels, *t* (1570.583) = 2.248, *p* = 0.025. There were no significant differences between the groups with respect to sex, *p* = 0.692, *DRD4*–7R, *p* = 0.478, and parent- and self-reported externalizing problems, *p* = 0.538 and 0.252, respectively.

### *DRD4*–7R and Chronic Stressors

Table 1 shows descriptive statistics and frequencies of the final sample and Table 2 Pearson correlations between predictors and parent-reported and self-reported EXTadj. There was no indication of gene-environment correlations as *DRD4*–7R was not significantly associated with chronic stressors at T2 (Spearman *r* = −0.03, *p* = 0.243) or T3 (*r* = 0.00, *p* = 0.932). See Online Resource 1 for number of chronic stressors reported and frequencies per chronic stressor.

**Table 1:** Descriptive statistics (left) and frequencies (right) of the variables used in this study

Variable		*N*	Mean (*SD*)	Range	0 (*N*)	1 (*N*)	2 (*N*)	3+ (*N*)
Age	T1	1621	11.09 (0.55)	10.01_12.58				
	T2	1620	13.35 (0.61)	11.58_15.08				
	T3	1576	16.14 (0.68)	14.42_18.48				
CBCL EXT^a^	T1	1573	6.03 (5.12)	0_31				
	T2	1586	4.62 (4.84)	0_29				
	T3	1451	4.46 (5.12)	0_34				
YSR EXT^a^	T1	1597	6.03 (4.41)	0_28				
	T2	1608	5.91 (4.20)	0_29				
	T3	1542	6.14 (4.52)	0_31				
Stressors^b^	T2	1587	1.27 (1.50)	0_10	657	389	256	285
	T3	1458	1.34 (1.57)	0_10	559	392	229	278
*DRD4*–7R^c^					1052	569		

*CBCL* Child Behavior Checklist, *YSR* Youth Self-Report, *EXT* Externalizing problems (DSM-oriented subscales oppositional defiant problems and conduct problems), *DRD4–7R* Dopamine D4 Receptor 7-repeat allele, *T* measurement wave

^a^Sum of 22 item scores for parent-report and 20 items for self-report; range per item 0–2

^b^Number of long-term difficulties experienced since previous measurement

^c^Coded as 0 = noncarrier; 1 = carrier

**Table 2:** Pearson correlation matrix of predictors and outcome variables, with parent-reported externalizing problems below and self-reported externalizing problems above diagonal

						Self-report	
	Variables	T2Stressors	T3Stressors	*DRD4*–7R^*a*^	Sex^*a*^	T2EXTadj	T3EXTadj
	T2Stressors	1	0.57***	-0.03	0.05	0.07**	0.06*
	T3Stressors	0.57***	1	0.00	-0.00	0.06*	0.09***
	*DRD4*–7R^*a*^	-0.03	0.00	1	0.07**	-0.01	-0.02
	Sex^*a*^	0.05	-0.00	0.07**	1	0.12***	0.16***
Parent-report	T2EXTadj	0.15***	0.15***	-0.02	0.09***	0.39***	0.31***
	T3EXTadj	0.13***	0.13***	-0.02	0.09***	0.30***	0.46***

*DRD4–7R* Dopamine D4 Receptor 7-repeat allele, *EXTadj* Externalizing problems adjusted for co-occurring internalizing problems, *T* measurement wave. *DRD4*–7R was coded as 0 = noncarrier; 1 = carrier. Sex was coded as 0 = female; 1 = male. ^*a*^Spearman rank order correlation, ***p < 0.001, **p < 0.01, *p < 0.05

**Fig. 1:**
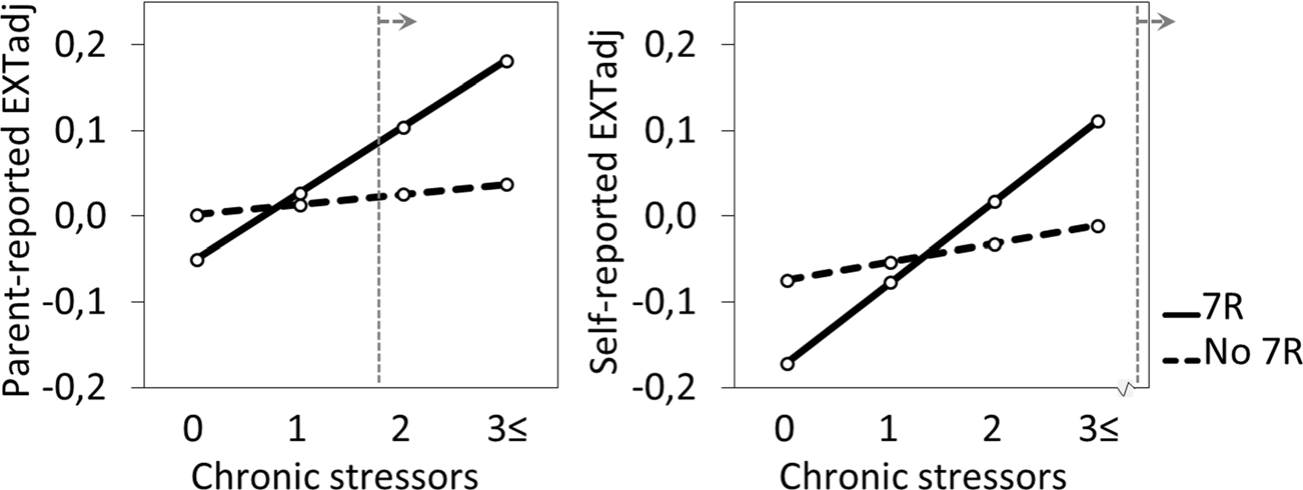
Adjusted externalizing problems reported by parents (left panel) and adolescents (right panel) increased significantly with number of chronic stressors in *DRD4*–7R carriers but did not change in noncarriers. *EXTadj* Externalizing problems adjusted for co-occurring internalizing problems, *7R* Dopamine D4 Receptor 7-repeat allele. Levels of chronic stressors refer to the number of long-term difficulties. According to post-hoc probing, the interaction effect is statistically significant on the right of the dashed line for parent-report (above 1.85) and outside our data range for self-report (above 4.80 stressors)

As shown in Table 3, parent-reported and self-reported EXTadj problems were significantly predicted by a two-way interaction effect of chronic stressors and *DRD4*–7R (*p* = 0.023 and *p* = 0.024, respectively). We plotted the levels of EXTadj for low, average, high, and very high levels of the truncated chronic stressor variable (corresponding to 0, 1, 2, and 3 or more long-term difficulties, respectively), separately for 7R carriers and noncarriers. Figure 1 shows that higher chronic stressor levels were related to higher EXTadj in 7R carriers, while EXTadj of noncarriers was stable across chronic stressor levels.

**Table 3:** The *DRD4*–7-repeat allele significantly interacted with chronic stressors level in predicting parent-reported and self-reported externalizing problems controlling for baseline externalizing problems

	Parent-reported EXTadj			Self-reported EXTadj		
Parameter	Estimate^a^	*SE* ^a^	*p*	Estimate^a^	*SE* ^a^	*p*
Intercept^b^	15.04	27.83	0.588	-50.36	31.34	0.108
Age	3.16	7.99	0.693	-5.96	9.26	0.520
Sex	-15.42	35.72	0.666	102.05	40.35	0.012
T1 EXTadj	594.31	18.07	<0.001	372.42	20.22	<0.001
Stressors	11.92	16.81	0.479	21.04	19.04	0.266
*DRD4*–7R	22.29	36.80	0.545	-14.54	40.93	0.725
*DRD4*–7R*Stressors	65.18	28.65	0.023	73.12	32.43	0.024

*EXTadj* Externalizing problems corrected for internalizing problems, *DRD4–7R* Dopamine D4 Receptor 7-repeat allele, *T* measurement wave. Variables were mean-centered except for *DRD4*–7R (0 = noncarrier; 1 = carrier) and sex (0 = female; 1 = male)

^a^Values multiplied by 1000 for ease of interpretation

^b^Participants varied significantly (*p <* 0.01) in intercept for parent-reported EXTadj, var.(u0j) = 263.97^a^, chi-square (1) = 240.66, and self-reported EXTadj, var.(u0j) = 316.20^a^, chi-square (1) = 207.23

Post-hoc probing of these interaction effects resulted in simple slopes and regions of significance. The increase in EXTadj with chronic stress level was statistically significant for 7R carriers, *t* (1557.022) = 3.85, *p* < 0.001 for parent-report, and *t* (1572.308) = 3.74, *p* < 0.001 for self-report, while the slope of EXTadj across chronic stress groups did not significantly differ from zero for noncarriers, *t* (1557.022) = 1.06, *p* = 0.288 for parent-report, and *t* (1572.308) = 1.41, *p* = 0.159 for self-report.

Regions of significance showed that the interaction effect between chronic stressors and *DRD4*–7R in predicting EXTadj was statistically significant below −1.34 and −0.32 chronic stressors for parent-report and self-report, respectively, both non-existent values, and above 1.85 and 4.80 chronic stressors for parent-report and self-report, respectively. We conclude that our findings apply to the upper end of the chronic stressor range, not to the lower end, and that the effect is stronger based on parent-report of externalizing problems than self-report.

Controlling for potential interaction effects of sex with chronic stressors or *DRD4*–7R, parent-reported and self-reported EXTadj problems were still significantly predicted by a two-way interaction effect of chronic stressors and *DRD4*–7R, *p* = 0.037 and *p* = 0.029, respectively. In these models, sex did not interact with *DRD4*–7R in predicting parent-reported or self-reported EXTadj, *p* = 0.524 and *p* = 0.241, respectively, nor with chronic stressors in predicting self-reported EXTadj, *p* = 0.150. However, sex did significantly interact with chronic stressors in predicting parent-reported EXTadj, *p* = 0.017, which may explain why the interaction effect of chronic stressors and *DRD4*–7R was somewhat weaker compared to our main results. Visual inspection showed that the association between chronic stressor levels and EXTadj was stronger in 7R carriers than in noncarriers (both boys and girls), as in Fig. 1, and stronger in boys than in girls (both 7R carriers and noncarriers). However, we found no evidence of a three-way interaction effect of chronic stressors, *DRD4*–7R, and sex, in predicting parent-reported or self-reported EXTadj, *p* = 0.610 and *p* = 0.251, respectively. These posthoc findings suggest that the association between chronic stressors level and EXTadj (at least parent-report) is moderated by *DRD4*–7R as well as by sex, but independent of each other.

Without adjusting externalizing for co-occurring internalizing problems, a two-way interaction effect of chronic stressors and *DRD4*–7R did not hold in predicting parent-reported EXT, *p* = 0.161, but still significantly predicted self-reported EXT, *p* = 0.045. Visual inspection showed that the association between chronic stressor levels and parent-reported EXT was strong overall with negligible differences between 7R carriers and noncarriers (albeit in the same direction as our main results). The association between chronic stressors level and self-reported EXT was similarly strong for 7R carriers but was attenuated in noncarriers, as in Fig. 1 but less pronounced. This weakening of effects due to co-occurring internalizing problems may suggest that our main findings pertain especially to “pure” externalizing problems and less to internalizing or comorbid externalizing and internalizing problems. In accordance, when we added internalizing problems as a covariate to the model, effects regained strength. Namely, a two-way interaction effect of chronic stressors and *DRD4*–7R was not significant in predicting parent-reported EXT, *p* = 0.099, but significant in predicting self-reported EXT, *p* = 0.029, approaching our original findings on externalizing problems adjusted for internalizing problems and with visual inspection showing similar plots as those depicted in Fig. 1. Thus, our main findings do not appear to be driven by the method we used to correct for internalizing problems.

These post-hoc analyses show the robustness and specificity of our main results. Tables and figures from these analyses are available upon request.

## Discussion

This study aimed to contribute to the literature by examining whether *DRD4*–7R moderated the association between chronic stressors and externalizing problems. As hypothesized, higher chronic stressor levels were related to higher externalizing levels in 7R carriers but not in noncarriers, suggesting high vs. low sensitivity, respectively, to adverse environments. These results were consistent across informants and were not driven by adolescents’ gender. Although it has been posited that the 7R allele reflects sensitivity to adverse as well as to beneficial environmental influences on externalizing problems (e.g., Bakermans-Kranenburg et al. 2008), this theory has received inconsistent support in adolescence (cf. Beach et al. 2010; Buil et al. 2015; Creemers et al. 2011; Janssens et al. 2015; Kretschmer et al. 2013; Marsman et al. 2013; Nederhof et al. 2012a; Nikitopoulos et al. 2014; Richards et al. 2015; Sonuga-Barke et al. 2009). In particular, with only one exception (Buil et al. 2015), none of these prior studies have convincingly demonstrated sensitivity to environmental influences in the adverse range. The present study thus adds to the literature by showing this sensitivity to adverse circumstances, at least to chronic stressors.

Our results contrast with prior findings that *DRD4*–7R did not moderate the association between early family adversity (a relatively broad measure of environmental influence, as the one used in the present study), and adolescents’ symptoms of CD/ODD and psychopathy (Nikitopoulos et al. 2014). These findings were based on data from a parent-interview, conducted when participants were 3 months old, assessing which of eleven family adversity factors (e.g., low educational level, marital discord) were present in the year prior to the child’s birth. One obvious explanation for the difference in findings would be the early age at which the environmental influence was assessed and, consequently, the large amount of time and contextual influences that passed between assessments of the environmental predictor and the behavioral outcome (i.e., 15 years), in contrast to our study, which assessed more recent environmental influences. However, given that the same study did show a moderating effect of *DRD4*–7R on the influence of laboratory-observed early maternal stimulation and responsiveness, also assessed at 3 months, we cannot conclude that *DRD4*–7R only moderates recent and not early environmental influences. The null finding may be due to prenatal family difficulties that are resolved before birth or that do not have longlasting effects on children. Moderating effects of *DRD4*–7R may be easier to detect when focusing on ongoing or chronic environmental difficulties, which presumably have a major impact on sensitive individuals, taxing their ability to cope, but not on less sensitive individuals.

Other prior findings that the 7R allele did not moderate adverse environmental influences on adolescents’ externalizing problems came from our own research group (Creemers et al. 2011; Kretschmer et al. 2013; Marsman et al. 2013; Nederhof et al. 2012a). These prior TRAILS findings have shown that the influences of parental rejection and overprotection, as perceived by preadolescents at T1 (mean age 11 years) on delinquency and aggression at T2 (mean age 13.5 years, combined parent-report and self-report; Marsman et al. 2013) and on substance use at T3 (mean age 16 years, self-report; Creemers et al. 2011) were not moderated by *DRD4*–7R (7R carriers vs. noncarriers). Furthermore, the influence of parental separation, assessed at T1 and T3, on self-reported externalizing levels at T3 did not differ between 7R carriers vs. noncarriers (Nederhof et al. 2012a). Finally, teacher-reported peer victimization at T2 did not influence self-reported delinquency at T4 (mean age 19 years) in 7R carriers, in contrast to 4R carriers (Kretschmer et al. 2013). These findings have led our colleagues to suggest that moderating effects of *DRD4*–7R on environmental influences on externalizing problems apply less to adolescence than to childhood (Kretschmer et al. 2013; Marsman et al. 2013), less to peer influence than to other environmental factors (Kretschmer et al. 2013), or may differ according to the operationalization of externalizing problems (Creemers et al. 2011). Given that samples, age range, and genetic and outcome measures used in these studies partially overlap with ours, it is likely that our findings differ due to the way in which we have operationalized environmental adversity. Whereas some of the previously addressed adversities may be ongoing, as were the difficulties we have assessed, our study appears to stand alone in its measurement of chronic difficulties that collectively capture many different aspects of individuals’ lives (e.g., both family and peer contexts). Thus, our findings suggest that moderating effects of *DRD4*–7R on the association between adverse environmental influences and externalizing problems do extend to adolescence when focusing on chronic multi-context stressors. However, this finding will need to be replicated by future research.

Although internalizing problems were not directly investigated in the current study, post-hoc findings suggest that interaction effects of *DRD4*–7R and stressors in predicting externalizing problems may be weakened by the presence of co-occurring internalizing problems. This is according to expectation, given that internalizing (unlike externalizing) problems lack a clear theoretical connection to *DRD4*–7R and given that prior studies found no evidence that *DRD4*–7R moderated the association between environmental influences and internalizing problems (Bakermans-Kranenburg and Van IJzendoorn 2006; maternal sensitivity; DiLalla et al. 2015; peer victimization).

Our study included a number of limitations. First, we collected parent-reports, not self-reports, of long-term difficulties because we assumed that parents are better and more stable judges of the difficulties that put chronic strain on family life. The stressors we examined included issues such as chronic housing problems and neighborhood problems. The drawback may be that such factors may be less stressful for adolescents than parents assume, thus overestimating stressor exposure. Another drawback of parent-report can be that parents may not have full insight into the other chronic stressors we measured, such as bullying, that weigh heavily on adolescents’ life, leading to underestimating stressor exposure. Although shared method variance may strengthen the interaction effect between chronic stressors and the 7-repeat allele in predicting externalizing problems reported by parents, it does not explain our similar findings on self-reported externalizing problems. Second, we focused on chronic adversities and did not study sensitivity to positive chronic conditions. A formal test of Differential Susceptibility includes both beneficial and adverse aspects of the environment, while we have only addressed the latter Diathesis-Stress model. Although sensitivity to beneficial environmental influences has been examined relatively frequently in relation to *DRD4–*7R, as outlined in the introduction, it certainly would have complemented our findings, had we been able to incorporate this.

Strengths of the study include the large sample of longitudinal, multi-informant data from pre-adolescence well into adolescence, large inter-individual differences in levels of externalizing problems and chronic stressors, and the use of Linear Mixed Modeling that allowed for optimal use of all available data from multiple measurements.

In sum, whereas previous studies on *DRD4*–7R as a moderator of environmental influences on adolescents’ externalizing problems have not convincingly demonstrated sensitivity to environmental influences in the adverse range, we were able to do so by focusing on chronic multi-context stressors. Our finding that higher levels of chronic stressors were associated with higher externalizing levels in 7R carriers but not in noncarriers suggests high vs. low sensitivity, respectively, to adverse environments. We encourage further studies of environmental influences that reflect multiple adverse aspects across multiple environmental domains (e.g., family, peers, school, and neighborhood).

## Electronic supplementary material

ESM 1 (DOCX 18 kb)

## References

[CR1] Achenbach TM, Rescorla LA (2001). Manual for the ASEBA school-age forms & profiles.

[CR2] Bakermans-Kranenburg MJ, Van IJzendoorn MH (2006). Gene-environment interaction of the dopamine D4 receptor (DRD4) and observed maternal insensitivity predicting externalizing behavior in preschoolers. Developmental Psychobiology.

[CR3] Bakermans-Kranenburg MJ, Van IJzendoorn MH (2011). Differential susceptibility to rearing environment depending on dopamine-related genes: new evidence and a meta-analysis. Development and Psychopathology.

[CR4] Bakermans-Kranenburg MJ, Van IJzendoorn MH, Pijlman FTA, Mesman J, Juffer F (2008). Experimental evidence for differential susceptibility: dopamine D4 receptor polymorphism (DRD4 VNTR) moderates intervention effects on toddlers' externalizing behavior in a randomized controlled trial. Developmental Psychology.

[CR5] Beach SRH, Brody GH, Lei MK, Philibert RA (2010). Differential susceptibility to parenting among African American youths: testing the DRD4 hypothesis. Journal of Family Psychology.

[CR6] Buil JM, Koot HM, Olthof T, Nelson KA, Van Lier PAC (2015). DRD4 genotype and the developmental link of peer social preference with conduct problems and prosocial behavior across ages 9-12 years. Journal of Youth and Adolescence.

[CR7] Chang FM, Kidd JR, Livak KJ, Pakstis AJ, Kidd KK (1996). The world-wide distribution of allele frequencies at the human dopamine D4 receptor locus. Human Genetics.

[CR8] Creemers HE, Harakeh Z, Dick DM, Meyers J, Vollebergh WAM, Ormel J (2011). DRD2 and DRD4 in relation to regular alcohol and cannabis use among adolescents: does parenting modify the impact of genetic vulnerability? The TRAILS study. Drug and Alcohol Dependence.

[CR9] De Winter AF, Oldehinkel AJ, Veenstra R, Brunnekreef JA, Verhulst FC, Ormel J (2005). Evaluation of non-response bias in mental health determinants and outcomes in a large sample of pre-adolescents. European Journal of Epidemiology.

[CR10] DiLalla LF, Bersted K, John SG (2015). Peer victimization and DRD4 genotype influence problem behaviors in young children. Journal of Youth and Adolescence.

[CR11] Dmitrieva J, Chen C, Greenberger E, Ogunseitan O, Ding YC (2011). Gender-specific expression of the DRD4 gene on adolescent delinquency, anger and thrill seeking. Social Cognitive and Affective Neuroscience.

[CR12] Ellis BJ, Boyce WT, Belsky J, Bakermans-Kranenburg MJ, Van IJzendoorn MH (2011). Differential susceptibility to the environment: an evolutionary-neurodevelopmental theory. Development and Psychopathology.

[CR13] Ferguson CJ (2015). Do angry birds make for angry children? A meta-analysis of video game influences on children's and adolescents' aggression, mental health, prosocial behavior, and academic performance. Perspectives on Psychological Science.

[CR14] Grant KE, Compas BE, Thurm AE, McMahon SD, Gipson PY (2004). Stressors and child and adolescent psychopathology: measurement issues and prospective effects. Journal of Clinical Child and Adolescent Psychology.

[CR15] Huisman M, Oldehinkel AJ, De Winter A, Minderaa RB, De Bildt A, Huizink AC (2008). Cohort profile: the Dutch ‘TRacking Adolescents’ Individual Lives’ Survey’; TRAILS. International Journal of Epidemiology.

[CR16] Janssens A, Van den Noortgate W, Goossens L, Verschueren K, Colpin H, De Laet S (2015). Externalizing problem behavior in adolescence: dopaminergic genes in interaction with peer acceptance and rejection. Journal of Youth and Adolescence.

[CR17] Jenkins JM, Rutter M, Bishop D, Pine D, Scott S, Stevenson J, Taylor EA, Thapar A (2008). Psychosocial adversity and resilience. Rutter's handbook of child and adolescent psychiatry.

[CR18] Keller MC (2014). Gene x environment interaction studies have not properly controlled for potential confounders: the problem and the (simple) solution. Biological Psychiatry.

[CR19] Kretschmer T, Dijkstra JK, Ormel J, Verhulst FC, Veenstra R (2013). Dopamine receptor D4 gene moderates the effect of positive and negative peer experiences on later delinquency: the TRacking Adolescents’ Individual Lives Survey study. Development and Psychopathology.

[CR20] Kwok OM, Underhill AT, Berry JW, Luo W, Elliott TR, Yoon M (2008). Analyzing longitudinal data with multilevel models: an example with individuals living with lower extremity intra-articular fractures. Rehabilitation Psychology.

[CR21] Marsman R, Oldehinkel AJ, Ormel J, Buitelaar JK (2013). The dopamine receptor D4 gene and familial loading interact with perceived parenting in predicting externalizing behavior problems in early adolescence: the TRacking Adolescents' individual lives survey (TRAILS). Psychiatry Research.

[CR22] Miller SA, Dykes DD, Polesky HF (1988). A simple salting out procedure for extracting DNA from human nucleated cells. Nucleic Acids Research.

[CR23] Nederhof E, Belsky J, Ormel J, Oldehinkel AJ (2012). Effects of divorce on Dutch boys’ and girls’ externalizing behavior in gene x environment perspective: diathesis stress or differential susceptibility in the Dutch TRacking Adolescents’ Individual Lives Survey study?. Development and Psychopathology.

[CR24] Nederhof, E., Jorg, F., Raven, D., Veenstra, R., Verhulst, F. C., Ormel, J., & Oldehinkel, A. J. (2012b). Benefits of extensive recruitment effort persist during follow-ups and are consistent across age group and survey method. The TRAILS study. *BMC Medical Research Methodology, 12*, 93. doi:10.1186/1471-2288-12-93. 10.1186/1471-2288-12-93 PMC3585928 22747967

[CR25] Nikitopoulos J, Zohsel K, Blomeyer D, Buchmann AF, Schmid B, Jennen-Steinmetz C (2014). Are infants differentially sensitive to parenting? Early maternal care, DRD4 genotype and externalizing behavior during adolescence. Journal of Psychiatric Research.

[CR26] Oldehinkel AJ, Verhulst FC, Ormel J (2008). Low heart rate: a marker of stress resilience. The TRAILS study. Biological Psychiatry.

[CR27] Ormel J, Oldehinkel AJ, Sijtsema J, Van Oort F, Raven D, Veenstra R (2012). The TRacking Adolescents’ Individual Lives Survey (TRAILS): design, current status, and selected findings. Journal of the American Academy of Child and Adolescent Psychiatry.

[CR28] Preacher KJ, Curran PJ, Bauer DJ (2006). Computational tools for probing interactions in multiple linear regression, multilevel modeling, and latent curve analysis. Journal of Educational and Behavioral Statistics.

[CR29] Propper C, Willoughby M, Halpern CT, Carbone MA, Cox M (2007). Parenting quality, DRD4, and the prediction of externalizing and internalizing behaviors in early childhood. Developmental Psychobiology.

[CR30] Ptacek R, Kuzelova H, Stefano GB (2011). Dopamine D4 receptor gene DRD4 and its association with psychiatric disorders. Medical Science Monitor.

[CR31] Richards JS, Hartman CA, Franke B, Hoekstra PJ, Heslenfeld DJ, Oosterlaan J (2015). Differential susceptibility to maternal expressed emotion in children with ADHD and their siblings? Investigating plasticity genes, prosocial and antisocial behaviour. European Child & Adolescent Psychiatry.

[CR32] Rutter M (2005). Environmentally mediated risks for psychopathology: research strategies and findings. Journal of the American Academy of Child and Adolescent Psychiatry.

[CR33] Savage J, Yancey C (2008). The effects of media violence exposure on criminal aggression - a meta-analysis. Criminal Justice and Behavior.

[CR34] Sonuga-Barke EJS, Oades RD, Psychogiou L, Chen W, Franke B, Buitelaar J (2009). Dopamine and serotonin transporter genotypes moderate sensitivity to maternal expressed emotion: the case of conduct and emotional problems in attention deficit/hyperactivity disorder. Journal of Child Psychology and Psychiatry.

[CR35] Verhulst FC, Van der Ende J (2013). Handleiding ASEBA. Vragenlijsten voor leeftijden 6 tot en met 18 jaar [ASEBA manual: questionnaires for ages 6 through 18 years].

[CR36] Windhorst DA, Mileva-Seitz VR, Linting M, Hofman A, Jaddoe VWV, Verhulst FC (2015). Differential susceptibility in a developmental perspective: DRD4 and maternal sensitivity predicting externalizing behavior. Developmental Psychobiology.

[CR37] Zandstra ARE, Hartman CA, Nederhof E, Van den Heuvel ER, Dietrich A, Hoekstra PJ, Ormel J (2015). Chronic stress and adolescents’ mental health: modifying effects of basal cortisol and parental psychiatric history. The TRAILS study. Journal of Abnormal Child Psychology.

[CR38] Zuckerman M (1999). Vulnerability to psychopathology: a biosocial model.

