# Supplementary material for: Chronic Stressors and Adolescents’ Externalizing Problems: Genetic Moderation by Dopamine Receptor D4. The TRAILS Study
Source: J Abnorm Child Psychol. 2017 Mar 30;46(1):73–82. doi: 10.1007/s10802-017-0279-4 (PMC5770493; doi:10.1007/s10802-017-0279-4)
Supplement: Supplementary file 1 — (DOCX 18 kb) [file 10802_2017_279_MOESM1_ESM.docx]

**Online Resource 1**

Number of chronic stressors reported and frequencies per chronic stressors.

This material is supplementary to:

Chronic stressors and adolescents’ externalizing problems: Genetic moderation by Dopamine Receptor D4. The TRAILS study. *Journal of Abnormal Child Psychology.*

**Table 1** Number of chronic stressors reported

|  |  | **T2** |  |  | **T3** |  |
| --- | --- | --- | --- | --- | --- | --- |
| **Stressors***^a^* |  | **n** | **%** |  | **n** | **%** |
| 0 |  | 657 | 41% |  | 559 | 38% |
| 1 |  | 389 | 25% |  | 392 | 27% |
| 2 |  | 256 | 16% |  | 229 | 16% |
| 3+ |  | 285 | 18% |  | 278 | 19% |
|  |  |  |  |  |  |  |
| 3 |  | 126 |  |  | 133 |  |
| 4 |  | 93 |  |  | 80 |  |
| 5 |  | 39 |  |  | 31 |  |
| 6 |  | 18 |  |  | 17 |  |
| 7 |  | 8 |  |  | 10 |  |
| 8 |  | 0 |  |  | 2 |  |
| 9 |  | 0 |  |  | 3 |  |
| 10 |  | 1 |  |  | 2 |  |

*^a^*Number of long-term difficulties experienced since previous measurement.

**Table 2** Frequencies per chronic stressor

|  | **T2** |  |  | **T3** |  |
| --- | --- | --- | --- | --- | --- |
| **Chronic stressor***^a^* | **n** | **%** |  | **n** | **%** |
| Chronic illnesses or physical handicaps of a family member | 375 | 24% |  | 363 | 25% |
| Lack of friends | 287 | 18% |  | 174 | 12% |
| High work pressure at school | 272 | 17% |  | 217 | 15% |
| Chronic illnesses or physical handicaps of the child | 260 | 16% |  | 209 | 14% |
| Being bullied | 254 | 16% |  | 132 | 9% |
| Long-lasting conflicts with family members | 111 | 7% |  | 196 | 13% |
| Long-lasting conflicts between family members | 105 | 7% |  | 144 | 10% |
| Long-lasting conflicts with others | 85 | 5% |  | 98 | 7% |
| Financial problems | 83 | 5% |  | 72 | 5% |
| Housing problems | 75 | 5% |  | 58 | 4% |
| Neighborhood problems | 54 | 3% |  | 30 | 2% |

*^a^*Specific long-term difficulties experienced since previous measurement.
